# Enhanced Visualization of Erythrocytes Through Photoluminescence Using NaYbF_4_:Yb^3+^,Er^3+^ Nanoparticles

**DOI:** 10.3390/bios15070396

**Published:** 2025-06-20

**Authors:** Vivian Torres-Vera, Lorena M. Coronado, Ana Patricia Valencia, Alejandro Von Chong, Esteban Rua, Michelle Ng, Jorge Rubio-Retama, Carmenza Spadafora, Ricardo Correa

**Affiliations:** 1 Biomedical Physics and Engineering Unit, Center of Cellular and Molecular Biology of Diseases (CBCMe), Instituto de Investigaciones Científicas y Servicios de Alta Tecnología (INDICASAT AIP), Panama City 1843-01103, Panama; viviator@ucm.es (V.T.-V.); lcoronado@indicasat.org.pa (L.M.C.); mng@indicasat.org.pa (M.N.); 2 Department of Chemistry in Pharmaceutical Sciences, Faculty of Pharmacy, Complutense University of Madrid, Plaza Ramon y Cajal 2, 28040 Madrid, Spain; bjrubio@ucm.es; 3 Nanobiology Group, Instituto Ramón y Cajal de Investigación Sanitaria (IRYCIS), Ctra. De Colmenar Viejo, Km. 9100, 28034 Madrid, Spain; 4 Sistema Nacional de Investigación (SNI), Secretaría Nacional de Ciencia, Tecnología e Innovación, Panama City 0816-02852, Panama; alejandro.von@utp.ac.pa; 5 Facultad de Ingeniería, Universidad Latina de Panamá, Panama City 0823-00933, Panama; anapatt1600@gmail.com; 6 School of Electrical Engineering, Universidad Tecnológica de Panamá, Víctor Levi Sasso Campus, Panama City 0819-07289, Panama; estebanruavilla@gmail.com

**Keywords:** rare-earth nanoparticles, red blood cells, photoluminescence, biocompatibility

## Abstract

Rare-earth nanoparticles (RE-NPs), particularly NaYF_4_:Yb^3+^,Er^3+^, have emerged as a promising class of photoluminescent probes for bioimaging and sensing applications. These nanomaterials are characterized by their ability to absorb low-energy photons and emit higher-energy photons through an upconversion luminescence process. This process can be triggered by continuous-wave (CW) light excitation, providing a unique optical feature that is not exhibited by native biomolecules. However, the application of upconversion nanoparticles (UCNPs) in bioimaging requires systematic optimization to maximize the signal and ensure biological compatibility. In this work, we synthesized hexagonal-phase UCNPs (average diameter: 29 ± 3 nm) coated with polyacrylic acid (PAA) and established the optimal conditions for imaging human erythrocytes. The best results were obtained after a 4-h incubation in 100 mM HEPES buffer, using a nanoparticle concentration of 0.01 mg/mL and a laser current intensity of 250–300 mA. Under these conditions, the UCNPs exhibited minimal cytotoxicity and were found to predominantly localize at the erythrocyte membrane periphery, indicating surface adsorption rather than internalization. Additionally, a machine learning model (Random Forest) was implemented that classified the photoluminescent signal with 80% accuracy and 83% precision, with the signal intensity identified as the most relevant feature. This study establishes a quantitative and validated protocol that balances signal strength with cell integrity, enabling robust and automated image analysis.

## 1. Introduction

Erythrocytes or red blood cells (RBCs) are differentiated anucleate cells with a unique morphology that facilitates the exchange of respiratory gases between the lungs and other body tissues [1]. The morphology of erythrocytes provide the maximum surface-area-to-volume ratio for optimal gas diffusion, and its structure contributes to the required degree of deformability, allowing erythrocytes to pass through the microvasculature [2]. The erythrocyte surface is negatively charged due to the abundance of sialic acid residues in its outer membrane [3]. This charge profile often produces electrostatic interactions with exogenous materials, which makes RBCs permeable to small, uncharged molecules like water, oxygen, carbon dioxide, glucose, and urea, but mostly impermeable to larger and charged molecules, including most proteins [4].

On the other hand, the interaction of surface-charged nanostructured materials with biomolecular systems is highly influenced by the physicochemical characteristics of the cell membrane [5]. In this context, erythrocytes represent a convenient and biologically relevant model for investigating nanoparticle–cell and nanoparticle–cell-surface interactions. Studying the behavior of erythrocytes in response to emerging nanomaterials is valuable in biomedicine as it provides fundamental insights into cell–material interface dynamics and may support the development of novel diagnostic strategies for diseases and conditions that directly affect erythrocytes and other diseases.

Currently, RE-NPs have gained popularity as luminescent probes for optical bioimaging [6,7]. Their distinct ability to absorb near-infrared (NIR) radiation and emit in the visible region of the spectrum via multiphoton upconversion processes mitigates the effects of cellular autofluorescence and phototoxicity in comparison with conventional fluorophores. In addition, RE-NPs enable live-cell imaging through their high photostability, sharp emission bands, and surface-functionalizable chemistry [8,9]. However, optimizing the photoluminescence response and obtaining reproducible imaging quality under biological conditions remains a major challenge [10,11].

## 2. Materials and Methods

The following reagents were bought from Sigma-Aldrich Inc. (Saint Louis, MO, USA) and used without further purification: ytterbium(III) chloride hexahydrate (99.9%) (YbCl_3_·6H_2_O), yttrium(III) chloride hexahydrate (99.9%) (YCl_3_·6H_2_O), erbium(III) chloride hexahydrate (99.9%) (ErCl_3_·6H_2_O), sodium hydroxide (NaOH) (≥98%), ammonium fluoride (NH_4_F) (≥98%), oleic acid (OA) (≥90% GC), 1-octadecene (ODE) (technical grade 90%), methanol (MeOH) (≥99.9% HPLC), absolute ethanol (EtOH), n-hexane (≥97% GC), poly-acrylic acid (PAA) (concentration = 50wt% in water; Mw: ~5000 g/mol), cyclohexane (≥97% GC), and hydrochloric acid (HCl) (37%). RPMI 1640, hypoxanthine, L-glutamine, gentamicin, glucose, and phenol red were purchased from Sigma-Aldrich and HEPES was acquired from WWR.

### 2.1. RBC In Vitro Culture

The blood used for the procedures was obtained from O^+^ donors after they provided informed consent for all the experiments. Briefly, CPDA-anticoagulated blood samples were centrifuged at 3000× *g* for 5 min at 4 °C. Following centrifugation, the plasma and buffy coats were carefully aspirated and, if not immediately processed, stored on ice. The resulting RBC pellet was resuspended in RPMI medium (Sigma-Aldrich) and washed three times using the same centrifugation parameters. After the final wash, the RBCs were resuspended in fresh RPMI medium to achieve a 50% hematocrit, which served as the stock suspension. All subsequent experiments utilized RBC suspensions adjusted to a final hematocrit of 2%.

### 2.2. Synthesis of NaYF_4_:Yb^3+^,Er^3+^ NPs

Monodisperse *β*-NaYF_4_:Yb^3+^,Er^3+^ nanoparticles were synthesized using the thermal co-precipitation method [12]. Initially, the salts YCl_3_·6H_2_O (0.78 mmol), YbCl_3_·6H_2_O (0.20 mmol), and ErCl_3_·6H_2_O (0.02 mmol) were dissolved in 1 mL of MeOH. Afterward, the solution was mixed with 1-octadecene (46.9 mmol) and oleic acid (19 mmol). The mixture was heated to 140 °C under a nitrogen flow using a heating mantle kept at the precise temperature by a dedicated controller. Subsequently, the HCl traces and solvents were removed with a vacuum pump. The next step involved adding 1 mL of a methanolic solution containing NaOH (2.5 mmol) and NH_4_F (4.0 mmol) and incubating for 30 min. The temperature was then increased to 110 °C under an N_2_ flow to remove the MeOH and H_2_O. Finally, the solution was heated to 316 °C and refluxed for 1 h. After allowing the solution to cool to RT, the nanoparticles were isolated and purified through aliquoting into four tubes and mixing with methanol. The distinct phases were divided, and the methanol portion was discarded. The sample was centrifuged at 8500 rpm for 15 min. The pellet was washed twice with 1 mL of EtOH without redispersing it. Finally, the pellet was dried and dispersed in 4 mL of hexane for storage.

### 2.3. Nanoparticle Surface Modification with Polyacrylic Acid

We followed the procedure for nanoparticle modification in [13]. Initially, 10 mg of NaYF_4_:Yb^3+^,Er^3+^ were distributed into two 1.5 mL tubes and centrifuged at 12,000 rpm for 10 min and the pellets were carefully dried to eliminate any remaining hexane. Subsequently, 1 mL of 0.1 M HCl was added to each tube, and the pellets were redispersed through sonication for 5 min in an ultrasonic bath. The dispersion was then gently shaken for 5 h. The RE-NPs were collected via centrifugation (10,000 rpm, 10 min), and the supernatant was discarded. The RE-NPs were redispersed in 1 mL of an aqueous PAA solution (*w*/*v*% = 2.5%, pH = 9.0). The reaction was left to incubate for 16 h under vigorous shaking at RT. RE-NPs-PAA were obtained through centrifugation at 12,000× *g* for 10 min. Then, the supernatant was discarded, and the nanoparticles were dispersed in 1 mL of MilQ ultra-pure water and centrifuged twice at 12,000× *g* for 10 min. Ultimately, the resulting pellet was redispersed in 250 μL of MilQ water.

### 2.4. Morphological Characterization

Transmission electron microscopy (TEM) images were recorded using a JEOL-1010 (Tokyo, Japan) transmission electron microscope operated at an acceleration voltage of 80 kV. The TEM sample was prepared by dropping 10 µL of the sample solution (3 mg/mL) onto a copper grid followed by solvent evaporation. To determine the crystalline phase, X-ray diffraction (XRD) patterns of the dried NP powders were obtained using a PANalytical Model XPPert PRO MPD Multi-Purpose Diffractometer (Almelo, The Netherlands). For nanoparticle characterization, especially to confirm the surface functionalization with PAA, a Thermo Nicolet 200IR spectrometer (Madison, WI, USA) was used to obtain the FT-IR spectrum. The samples were prepared by grinding the dried nanoparticles with potassium bromide (KBr), which were then processed into a pellet using a mechanical press. Air and moisture were removed using a vacuum pump. Background and sample spectra were recorded in absorption mode using a resolution of 4 cm^−1^ and 128 scans. The TGA employed a TGA/DSC 1STAR system (Mettler Toledo, Columbus, OH, USA). The samples (~5 mg NPs) were dried in a 100 μL alumina crucible at 82 °C. The experiment involved a heating program up to 530 °C, using gas flows of 20 cm^3^/min for O_2_ and N_2_. Z-potential measurements were performed using a Malvern Nano-ZS instrument. The samples were freshly prepared (50 μg/mL) and the measurements were conducted at 25 °C using the automatic mode, an equilibration time of 120 s, and the Smoluchowski fit model, with at least three independent measurements made.

### 2.5. Optical Characterization

The luminescence spectra of the emission of the NPs-RE were measured using a fluorescence system that was built in the optics laboratory of the MatNaBio research group at the Complutense University of Madrid. The apparatus used for the photoluminescence measurements operates as follows: the excitation beam comes from a 10 W continuous wave (CW) laser (L4-9897603, JDS Uniphase Corporation, Chandler, AZ, USA) operating at a wavelength of 976 nm and is equipped with a current and temperature controller ((ILX Lightwave, LDX-36025-12 and LDT-5525B, Newport Corporation, Irving, CA, USA). The laser beam passes through a short-pass dichroic filter FF01-775/SP (Semrock, Rochester, NY, USA) and is then focused with a 10× objective into a Hellma 101.015-QS microcuvette (3 × 3 mm^2^ thick, Hellma Müllheim, Germany). The sample luminescence is reflected by the dichroic mirror towards the short-pass filter, which blocks reflected radiation between 770 and 1050 nm. Three spectra were recorded using a Tmc300 monochromator coupled with a DH 30 TE cooled multi alkali PMT (Bentham, North Yorkshire, UK). The intensity was calculated by integrating the area of the spectra within the emission bands and taking the maximum deviation as the error.

### 2.6. Cellular Uptake and Cytotoxicity Studies with Vero Cells

Cellular viability was performed using the standard 3-(4,5- dimethyl-2-thiazolyl)-2,5-di-phenyl-2-H-tetrazolium bromide (MTT) assay method to evaluate the biocompatibility of the RE-NPs [14]. For this bioassay, 5000 cells per well were seeded together with varying concentrations of NPs-PAA (0.1–5 mg/mL) in a total volume of 100 μL per well. Subsequently, the cells were incubated for 72 h at 37 °C. Briefly, the culture medium was removed, and the cultures were washed twice with a phosphate-buffered saline solution (PBS 1X). Typically, 100 μL of cell culture medium and 25 μL of MTT reagent were added to each well and incubated at 37 °C for 4 h. When the purple precipitate of formazan crystals was clearly visible under a microscope, 100 μL of isopropanol was added to each well until the purple crystals completely dissolved. Absorbance at 550 nm was measured using a BioTek Synergy HT plate reader (Winooski, VT, USA). The cell viability was expressed as a percentage of that of the control, which was set at 100%. All the results are the averages ± SD of three samples.

### 2.7. Hemolysis Bioassay

The hemolytic activity of the NaYF_4_:Yb^3+^,Er^3+^@PAA NPs was measured using the hemolysis bioassay reported in [15]. Different concentrations of NPs@PAA were prepared in the range of 0.1 to 5.0 mg/mL, using RBCs as a negative control and Triton X-100 (0.01 M) (Saint Louis, MO, USA), a strong hemolytic agent, as a positive control. In a 96-well plate, 180 µL of the 2% RBC suspension in blood and the different concentrations of NPs were added. The plate was incubated at 37 °C with a CO_2_ flow for 24 h. After 24 h, the plate was centrifuged at 2000 rcf for 5 min; a 160 µL aliquot of the supernatant was taken, and the absorbance was measured at a wavelength of 415 nm using a BioTek Synergy HT multimode plate reader (Winooski, VT, USA).

### 2.8. Colocalization of NaYF_4_:Yb^3+^,Er^3+^ Nanoparticles in Erythrocytes

Different experimental conditions were evaluated to optimize the detection and interaction of NaYF_4_:Yb^3+^,Er^3+^ -NPs with erythrocytes. RBC samples at 2% hematocrit were incubated with RE-NPs dissolved in 100 mM HEPES solution at several concentrations, with a final volume of 300 µL per sample. The incubations were performed at 37 °C in a CO_2_ incubator set to 5% CO_2_ for defined periods of time. After incubation, the samples were washed with 100 mM HEPES and centrifuged at 2000 rcf to remove any excess RE-NPs. Subsequently, the samples were exposed to different current intensities using a CLD1015 (Thorlabs, Newton, NJ, USA) 980 nm laser, and fluorescent images were acquired with an Olympus IX70 fluorescence microscope equipped with a CMOS camera (Orca-flash 4.0, Hamamatsu, Shizuoka, Japan). To optimize the fluorescent intensity, the signal-to-noise ratio and the quality of the erythrocyte image labeled with NaYF_4_:Yb^3+^,Er^3+^ nanoparticles under the various conditions were evaluated, as described in Table 1.

### 2.9. Processing and Analysis of Images After Acquisition

To assess the presence of photoluminescent signals, even weak signals, from erythrocyte-labeled rare-earth nanoparticles, a supervised machine learning approach was utilized. Regions of interest (ROIs) were manually annotated a priori based on bright field images and transposed to their corresponding photoluminescence images. Each ROI was annotated as either positive or negative based on the presence of a detectable photoluminescent signal through direct visualization. Additional background ROIs not containing erythrocytes were added as negative controls to aid model generalization. A set of descriptors was extracted from each pixel in the ROI including area, mean intensity, minimum and maximum intensities, integrated density, and density of pixels using the Fiji software 2.9.

The final dataset, with labeled ROI measurements, was transferred into Python 3.12 (2023). All quantitative features were z-score standardized before evaluating. The labeled dataset was then randomly split into stratified training (70% of data) and test (30% of data) subsets to maintain the same proportion of positive to negative samples. Standardized data were used to train a Random Forest classifier to predict whether a photoluminescent signal was present or absent. The performance of the model was evaluated as a composite metric of accuracy, precision, recall, F1-score, and area under the receiver operating characteristic curve (AUC-ROC).

Feature importance was assessed using the trained Random Forest model to determine features ranked according to their contribution to the classification. The performance metrics were calculated using the test set data. A confusion matrix was generated to visualize the classification performance. An ROC curve was plotted to evaluate the ability of the classifier to discriminate between the classes as the discrimination threshold was changed. The current workflow provides an automatable, reproducible, and scalable pipeline for the detection of nanoparticle signals in erythrocyte imaging datasets and allows for objective validation of photoluminescence under variable imaging conditions.

## 3. Results

### 3.1. Characterization of NaYF_4_:Yb^3+^, Er^3+^- NPs

**Figure 1:**
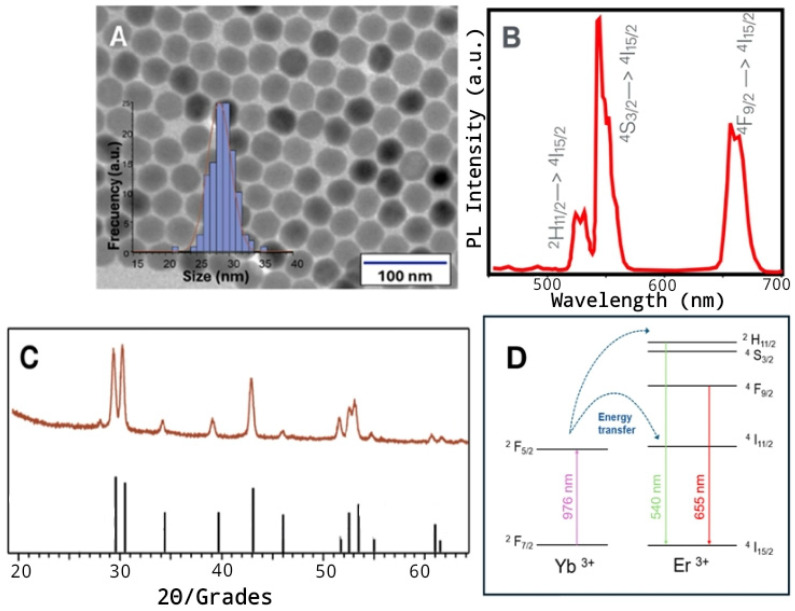
(**A**) Transmission electron microscopy (TEM) image of the synthesized NaYF_4_:Yb^3+^/Er^3+^ NPs. The inset shows the particle size distribution. (**B**) Photoluminescence (PL) spectrum of NaYF_4_:Yb^3+^,Er^3+^ NPs under excitation at 980 nm, (**C**) X-ray diffraction (XRD) pattern of NaYF_4_:Yb^3+^,Er^3+^ -NPs and reference (JCPDS card 28-1192) for the hexagonal β-phase of NaYF_4_. (**D**) Representative scheme for the mechanism of upconversion photoluminescence by energy transfer.

The morphological and structural characteristics of the synthesized NaYF_4_:Yb^3+^,Er^3+^ nanoparticles (NPs) were thoroughly investigated prior to biological application. The transmission electron microscopy (TEM) analysis revealed the formation of highly homogeneous nanoparticles with a uniform hexagonal shape. Quantitative analysis of the TEM images indicated a narrow size distribution, with an average particle diameter of 29 ± 3 nm (Figure 1A). The optical properties of the synthesized nanoparticles were studied by photoluminescence (PL) spectroscopy. Upon illumination with a 980 nm CW laser, the NPs depicted the characteristic upconversion luminescence spectrum associated with the Er^3+^ dopant ions. This upconversion process transforms low-energy excitation photons into visible light emission. It relies on the ability of Yb^3+^ ions to absorb low-energy photons at a wavelength of 980 nm, thereby populating their ^2^F_5/2_ energy level. The absorbed energy is then transferred through multiple steps to the Er^3+^ emitter ions (Figure 1B), leading to the sequential population of their excited energy levels and resulting in an upconverting emission. This spectrum was dominated by intense green emission bands observed between 520 nm and 550 nm, specifically corresponding to the ^2^H_11/2_→^4^I_15/2_ and ^4^S_3/2_→^4^I_15/2_ electronic transitions within the erbium ions. Additionally, a distinct red emission band centered at 650 nm, arising from the ^4^F_9/2_→^4^I_15/2_ transition, was clearly visible. Further structural confirmation was obtained via X-ray diffraction (XRD). The diffraction pattern obtained from the NP powder sample exhibited sharp peaks that perfectly match the standard reference pattern for the hexagonal β-phase crystal structure of NaYF_4_ (JCPDS Card No. 28-1192). No diffraction peaks corresponding to other crystalline phases (e.g., cubic α-phase) or impurities were detected, confirming the phase purity of the synthesized material (Figure 1C).

The surface of the synthesized oleate-capped nanoparticles was successfully modified via ligand exchange to introduce a hydrophilic polyacrylic acid (PAA) coating, a step crucial for enhancing their stability and dispersibility in aqueous biological media. Fourier-transform infrared spectroscopy (FT-IR) provided chemical evidence for the successful surface functionalization. Comparison of the spectra before and after modification showed the disappearance of bands associated with oleic acid and the emergence of new bands characteristic of PAA. Notably, a broad band around 3446 cm^−1^ indicated O–H stretching vibrations, while the region between 1400 and 1800 cm^−1^ contained key signatures: a band at 1480 cm^−1^ (C–O stretching); strong bands at 1560 cm^−1^ and 1457 cm^−1^ that represent the asymmetric and symmetric stretching vibrations of carboxylate anions (–COO–), respectively; and a band at 1720 cm^−1^ assigned to the C=O stretching of free carboxylic acid groups (–COOH) within the PAA polymer chains. The prominent carboxylate bands suggest effective chelation of the PAA to the nanoparticle surface via coordination with surface lanthanide (Y^3+^, Yb^3+^, and Er^3+^) ions (Figure S1A). The change in surface chemistry was further corroborated by the zeta potential measurements, which yielded a significantly negative value of approximately −30 mV in an aqueous suspension. This negative potential confirmed the presence of carboxylate groups due to the PAA coating, contributing to electrostatic repulsion and thus enhancing colloidal stability. Thermogravimetric analysis (TGA) was employed to assess the NPs’ thermal stability and quantify the organic coating content. The TGA curve displayed distinct weight loss stages: an initial loss of about 5% between 50 °C and 200 °C, which was attributed to the desorption of physically adsorbed water and residual solvents; a major weight loss occurring between 200 °C and 400 °C, corresponding to the thermal decomposition of the PAA polymer layer; and a more gradual weight loss between 400 °C and 600 °C, likely due to the degradation of carbonaceous residues. The substantial remaining mass percentage of approximately 85% at the end of the heating program confirmed the presence of the thermally stable inorganic NaYF_4_ core (Figure S1B).

### 3.2. Viability Bioassays

**Figure 2:**
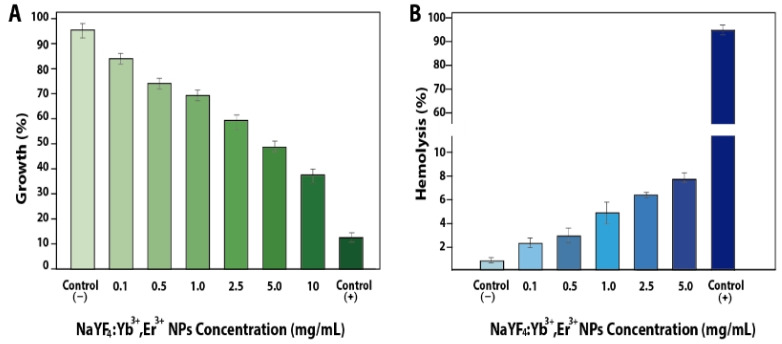
(**A**) MTT assay results showing the viability of Vero cells exposed to different concentrations of NaYF_4_:Yb^3+^,Er^3+^@PAA NPs. The negative control corresponds to untreated Vero cells, and the positive control is doxorubicin. (**B**) Hemolysis assay results for RBCs at 2% hematocrit exposed to different concentrations of NaYF_4_:Yb^3+^,Er^3+^@PAA NPs. The negative control corresponds to untreated erythrocytes, while the positive control is 0.1 M Triton X-100.

To evaluate the suitability of the NaYF_4_:Yb^3+^,Er^3+^@PAA NPs for biological applications, their potential toxicity was assessed using standard in vitro assays. Cytotoxicity was measured using the MTT assay on Vero cell cultures, a commonly used mammalian cell line for cytotoxic studies. The results clearly indicated a dose-dependent effect on cell viability after 72 h of exposure. At the lowest concentration tested (0.1 mg/mL), the cell viability remained high, approximately 90%, relative to the untreated control cells. However, as the NP concentration increased, the viability progressively decreased, to about 80% at 0.5 mg/mL, 75% at 1.0 mg/mL, 66% at 2.5 mg/mL, and dropped markedly to around 40% at the highest concentration of 10 mg/mL. The positive control, doxorubicin, induced almost complete cell death (~10% viability) (Figure 2A). The hemolytic potential of the NPs was specifically evaluated using freshly isolated human erythrocytes (RBCs) incubated at 2% hematocrit for 24 h. Hemolysis, quantified by measuring the amount of hemoglobin released into the supernatant, was minimal (~2%) at an NP concentration of 0.1 mg/mL. A moderate, concentration-dependent increase in hemolysis was observed, reaching 5% at 0.5 mg/mL, approximately 10% at 1 mg/mL, 15% at 2.5 mg/mL, and peaking at 20% at 5 mg/mL (Figure 2B). Importantly, even at the highest concentration tested, the hemolysis induced by the NPs was significantly lower than that caused by the positive control, 0.1 M Triton X-100 detergent, which resulted in nearly complete (98%) RBC lysis. Considering both the cytotoxicity and hemolysis results, the subsequent imaging experiments were conducted using NP concentrations at or below 1.0 mg/mL to ensure minimal perturbation of cell health and membrane integrity while still providing a sufficient signal for detection.

### 3.3. In Vitro Fluorescence Imaging of Human Erythrocytes Using NaYF_4_:Yb^3+^, Er^3+^ NPs

**Figure 3:**
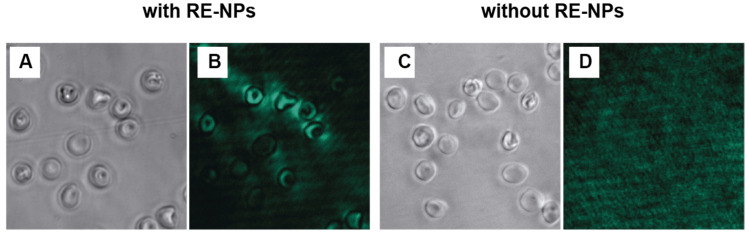
Photoluminescence imaging of erythrocytes incubated with and without rare-earth nanoparticles (NaYF_4_:Yb^3+^,Er^3+^@PAA-NPs). Bright-field image of erythrocytes (**A**) incubated with RE-NPs and (**B**) after excitation with a 980 nm laser. Bright-field image of (**C**) control erythrocytes without RE-NPs and (**D**) after 980 nm laser excitation. All fluorescence images were acquired with a 200 ms exposure time.

Optimization of the experimental parameters was performed to maximize the quality and reliability of the photoluminescence images acquired from human erythrocytes incubated with RE-NPs@PAA. Figure 3 shows the photoluminescence images of erythrocytes incubated with RE-NPs (Figure 3A) and pristine erythrocytes that were not incubated with RE-NPs (Figure 3B). Upon excitation with a 980 nm laser, the erythrocytes incubated with RE-NPS emitted light and revealed a morphology closely resembling that observed in the bright-field images. In contrast, the erythrocytes not incubated with RE-NPs did not exhibit any visible emission after illumination with the 980 nm CW laser. This experiment confirmed the interaction between the RE-NPs and the erythrocytes and the specificity of the upconverting emission.

#### 3.3.1. HEPES Concentration

**Figure 4:**
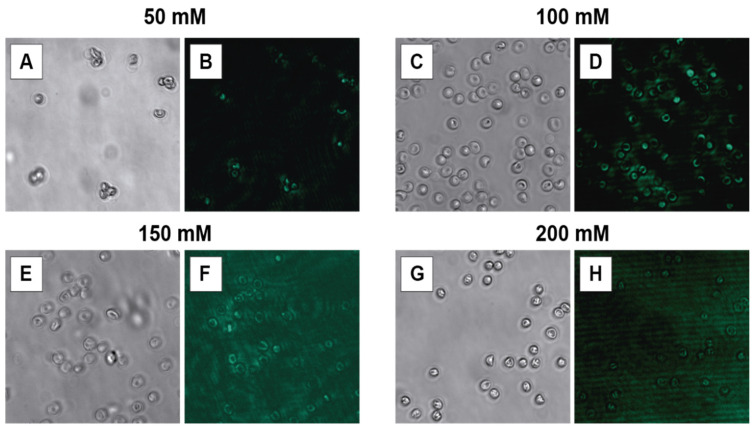
Micrographs of 2% erythrocytes after being incubated for 3 h with NaYF_4_:Yb^3+^,Er^3+^@PAA NPs 0.01 mg/mL. Images (**A**,**C**,**E**,**G**) were obtained using a bright field microscope, while images (**B**,**D**,**F**,**H**) show the photoluminescence (green) of the nanoparticles after excitation with a 980 nm laser. All fluorescence images were acquired with a 200 ms exposure time.

The choice of suspension buffer is critical for maintaining erythrocyte integrity and preserving the NPs’ optical properties. Four different concentrations of HEPES buffer (50, 100, 150, and 200 mM, all adjusted to pH 7.2) were tested. Bright-field microscopy revealed significant erythrocyte lysis when suspended in 50 mM HEPES, indicating an insufficient ionic strength (Figure 4A,B). In contrast, the cell morphology appeared to be preserved at 150 mM and 200 mM HEPES. On the other hand, under these conditions, the photoluminescence images suffered from notably increased background autofluorescence, which negatively affected the specific emission arising from the nanoparticles and diminished the overall signal-to-noise ratio, thereby reducing the image quality (Figure 4F,H). The optimal condition was found to be 100 mM HEPES buffer, which effectively maintained the characteristic biconcave morphology of the erythrocytes without inducing lysis and provided a strong, clear photoluminescence signal from the associated nanoparticles with minimal background interference (Figure 4C,D).

#### 3.3.2. Incubation Time

**Figure 5:**
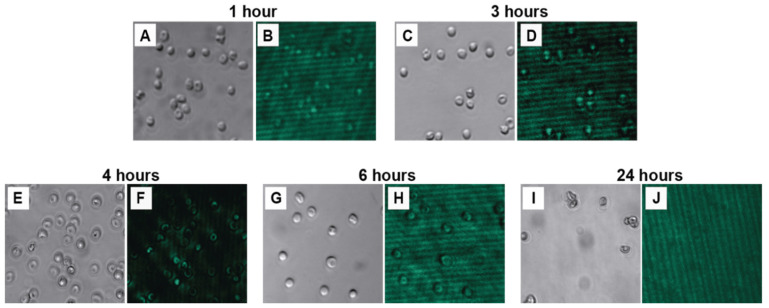
Photoluminescence images (green) of 2% erythrocytes incubated with NaYF4:Yb^3+^,Er^3+^@PAA nanoparticles (0.01 mg/mL) in HEPES buffer (100 mM) under near-infrared excitation (980 nm) at different incubation times (1, 3, 4, 6, and 24 h). The images corresponding to each time point are presented in pairs: bright-field images (**A**,**C**,**E**,**G**,**I**) and the corresponding photoluminescence images (**B**,**D**,**F**,**H**,**J**). All fluorescence images were acquired with a 200 ms exposure time.

The duration of incubation determines the extent of the nanoparticle–cell interactions. Erythrocytes were incubated with 0.01 mg/mL NPs in 100 mM HEPES for varying periods (1, 3, 4, 6, and 24 h). The assessment via bright-field microscopy indicated that the native biconcave disc shape of the erythrocytes was largely maintained under incubation times of up to 4 h (Figure 5A,C,E). However, subtle morphological alterations, such as contoured surfaces, began to appear after 6 h of incubation (Figure 5G), and significant deviations from normal morphology, including irregular shapes, were evident after 24 h (Figure 5I). Quantitative analysis of the corresponding photoluminescence images involved measuring average signal intensity (brightness) and signal definition (edge density). The analysis revealed that the average signal intensity peaked after 4 h of incubation (brightness score: 218.17 a.u.), suggesting that this duration allowed for the maximum association or uptake of NPs by the cells (Table S1, Figure 5F). Shorter incubation times (1 and 3 h) resulted in considerably weaker signals (Figure 5B,D), while longer times (6 and 24 h) showed a marked decrease in signal intensity, potentially due to NP degradation or release, or cell damage (Table S1, Figure 5H,J). Although the signal definition was highest at 6 h, the concurrent drop in intensity made the 4-h time point the most favorable compromise, offering the brightest signal while preserving cell integrity.

#### 3.3.3. Laser Current Intensity

**Figure 6:**
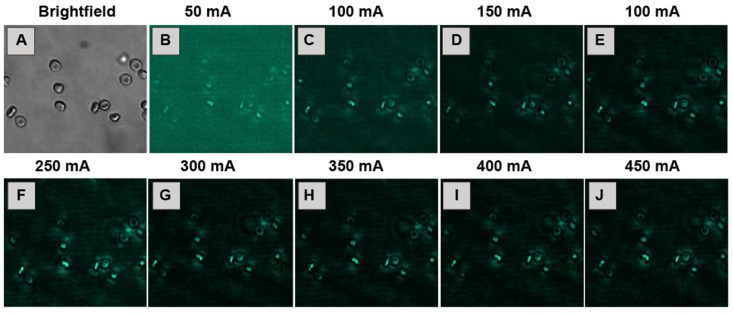
Images of erythrocytes at 2% hematocrit incubated for 4 h with NaYF_4_:Yb^3+^,Er^3+^@PAA NPs (0.01 mg/mL) and 100 mM HEPES (pH 7.2). Image (**A**) was obtained using a bright-field microscope. Images (**B**–**J**) show the effect of increasing the laser current intensity on the photoluminescence signal (green) from the nanoparticles after excitation with a 980 nm laser. All fluorescence images were acquired with a 200 ms exposure time.

The intensity of the 980 nm excitation laser directly influences the upconversion emission brightness. The laser current intensity, controlled via the applied current, varied from 50 mA up to 450 mA. At low intensity settings (50–200 mA), the resulting photoluminescence signal from the nanoparticles associated with erythrocytes was found to be weak and was often difficult to distinguish clearly from the background (Figure 6B–E), suggesting an insufficient excitation energy to efficiently populate the excited states of the Er^3+^ ions.

As the current intensity increased into the intermediate range of 250–300 mA, the emission signal intensity rose substantially, yielding bright images with good contrast between the labeled cells and the background (Figure 6F,G). This range appeared to be optimal for balancing signal strength and image quality. However, increasing the current intensity to higher values (350–450 mA) led to apparent saturation of the detector or the upconversion process itself, resulting in overly bright images where cellular details were lost and increased light scattering became evident, compromising image definition (Figure 6H–J). Therefore, the optimal excitation laser current intensity range was identified as 250–300 mA.

#### 3.3.4. NP Concentration

**Figure 7:**
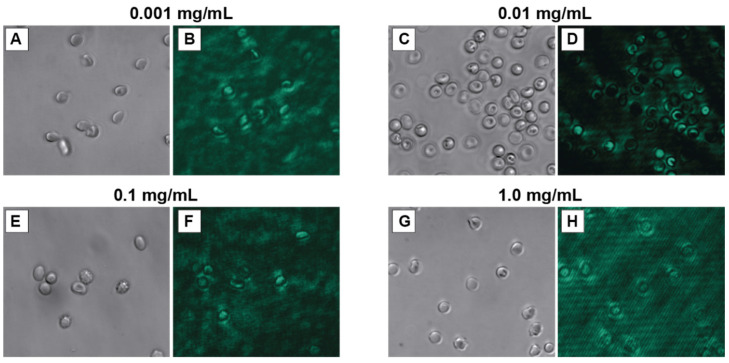
Representative images of erythrocytes after exposure to different concentrations of NaYF_4_:Yb^3+^,Er^3+^@PAA nanoparticles, obtained by bright-field microscopy (**A**,**C**,**E**,**G**) and PL microscopy after excitation with a 980 nm laser (**B**,**D**,**F**,**H**). Images were acquired at a laser current intensity of 300 mA and incubation time of 4 h. All fluorescence images (green) were acquired with a 200 ms exposure time.

The concentration of the NPs affected both the signal intensity and potential biological effects. Taking this effect into account, erythrocytes were incubated (4 h, 100 mM HEPES) with varying RE-NP concentrations ranging from 0.001 to 1.0 mg/mL. Even at the lowest concentration tested (0.001 mg/mL), a faint but discernible photoluminescence signal could be detected, primarily localized around the periphery of the erythrocytes (Figure 7B). Increasing the concentration to 0.01 mg/mL resulted in a significantly brighter and clearer signal, again predominantly observed along the cell edges, while the bright-field images indicated that most of the erythrocytes retained their normal morphology (Figure 7C,D). Conversely, at higher concentrations of 0.1 mg/mL and 1.0 mg/mL, while the overall signal was intense, it often appeared more diffuse and heterogeneous across the cell populations. Crucially, these higher concentrations induced noticeable morphological changes in the erythrocytes, including a shift towards spherical shapes (spherocytosis), surface deformations, and the formation of cell aggregates, suggesting significant membrane perturbations (Figure 7E–H). A separate test confirmed that a detectable signal, albeit weak, could still be obtained at a concentration even below 0.0001 mg/mL. To achieve a strong signal with minimal morphological impact, 0.01 mg/mL was determined to be the optimal working concentration.

#### 3.3.5. Laser Exposure Time

**Figure 8:**
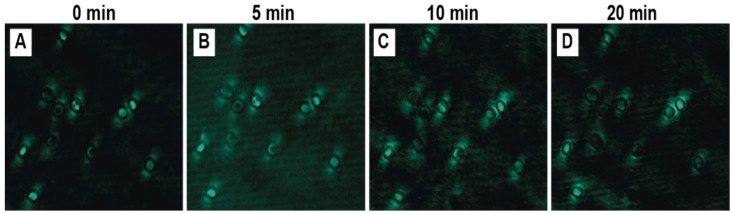
Photoluminescence images (green) of erythrocytes at 2% hematocrit after incubation for 4 h with NaYF_4_:Yb^3+^,Er^3+^@PAA NPs (0.01 mg/mL). The images correspond to different exposure times to excitation with a 980 nm laser: (**A**) 0 min, (**B**) 5 min, (**C**) 10 min, and (**D**) 20 min. All fluorescence images were acquired with a 200 ms exposure time.

The photostability of the upconversion signal under continuous laser irradiation (980 nm, 300 mA) was assessed by acquiring images after exposure times of 0, 5, 10, and 20 min. The initial photoluminescence signal (0 min) was bright and well-defined (Figure 8A). After 5 min of continuous exposure, the signal intensity and quality remained largely unchanged, indicating good stability over this period (Figure 8B). However, a noticeable decline in brightness and sharpness became apparent after 10 min of exposure (Figure 8C), and this degradation was more pronounced after 20 min, with the signal appearing significantly weaker and more diffuse (Figure 8D). This suggests that prolonged exposure can lead to photobleaching of the nanoparticles or could potentially induce photothermal stress in the cells, affecting signal integrity. Consequently, it was determined that image acquisition times should ideally be shorter than 10 min to ensure signal fidelity.

#### 3.3.6. Machine Learning-Based Analysis of Erythrocyte Photoluminescence

**Figure 9:**
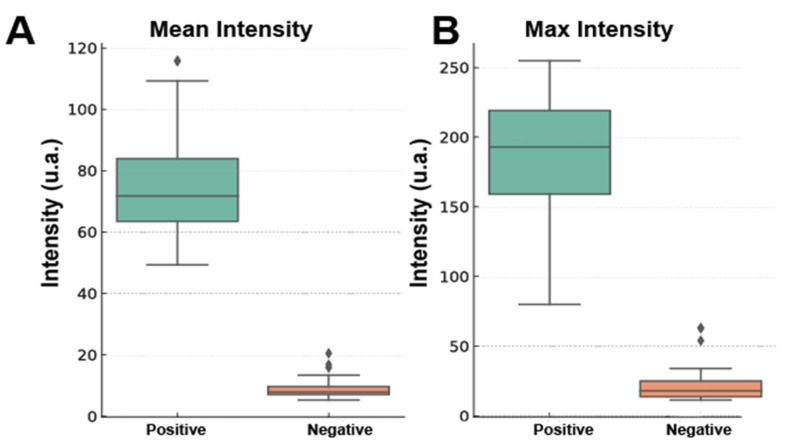
Boxplots showing the distribution of (**A**) mean intensity and (**B**) maximum intensity.

A total of 109 annotated regions of interest (ROIs) were analyzed, including regions with erythrocytes with a photoluminescent signal (positive, n = X) vs. regions with erythrocytes or background regions without a signal (negative, n = Y). The following quantitative descriptors were extracted from each ROI: area, mean intensity, maximum intensity, integrated density (IntDen), and pixel density. Figure 9 and Figure S2 show the results.

The descriptive analysis based on the intensity features showed clear quantitative differences between the ROIs classified as being positive and negative for photoluminescent signals. Figure 9 shows that the positive ROIs presented a significantly higher mean intensity (mean ± standard deviation (SD) = 74.22 ± 14.03) than the negative ROIs (9.13 ± 3.32), with medians of 71.74 and 7.85, respectively. The trend of the maximum intensity was consistent with that of the mean intensity, with the positive group displayed considerably higher values (mean = 188.73; max = 255.0) than the negative group (mean = 22.63; max = 63.0).

The integrated density metric takes into account both the intensity and the size of the ROI and showed the clearest separation, with positive ROIs having a mean integrated density of 483.199 (median: 471.794), whereas negative ROIs had a mean integrated density of 52.296 (median: 47.371). These findings highlight the value of discriminating based on pixel intensity features and confirmed that NaYF_4_:Yb^3+^,Er^3+^ photoluminescent labeling yields a strong and measurable signal that is readily distinguished by automated analysis.

A Random Forest classifier was trained on five normalized features (area, mean, min, max, and pixel density) to classify the presence of a photoluminescent signal in erythrocyte ROIs. The data was randomly split into training and test sets (0.7:0.3) while preserving a stratified distribution of positive and negative cases. The model exhibited strong classification performance on the test set, achieving an overall accuracy of 92% and class-specific precision, recall, and F1-score of 80%, 83%, and 81%, respectively. The area under the receiver operating characteristic curve (AUC-ROC) was 0.85, indicating a good ability to discriminate between the classes (Figure S3). These results suggest that the classifier can reliably identify ROIs with detectable photoluminescent signals, even within the context of potential imaging variability or subtle signal strengths.

**Figure 10:**
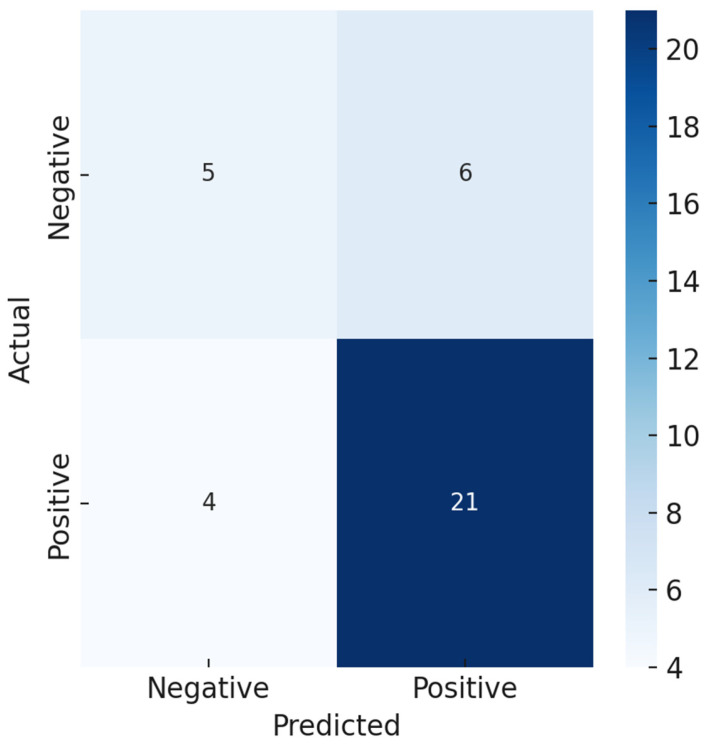
Confusion matrix illustrating the classification performance of the Random Forest model on the test set. The matrix shows the number of true positives (21), true negatives (5), false positives (6), and false negatives (4).

In the confusion matrix (Figure 10), the number of false positives (FPs) and false negatives (FNs) was low, showing that the model performed equally well for both classes. The high recall (sensitivity) score means that most true positive ROIs were detected by the classifier. The high precision score indicates that a positive prediction was highly likely to represent a true signal. Our findings demonstrate that this supervised machine learning method may facilitate semi-automated analysis of live-cell photoluminescent nanoparticle–cell interactions.

Based on the Random Forest model, we evaluated the relative importance of each feature utilized in the classification. Every feature was assigned an importance score, which represented the contribution of each feature in the model’s decision-making process and was averaged over all decision trees within the ensemble. In order, the top three most important predictors were the maximum intensity, the mean intensity, and the integrated density (IntDen). These features collectively captured both the intensity and the spatial extent of the photoluminescent signal within each ROI. The maximum intensity proved to be the most informative feature in this analysis, likely due to its ability to capture bright and highly localized photoluminescent hotspots in the positive ROIs. The mean intensity, summarizing the photoluminescent signal strength across the ROI, and IntDen, which incorporates both the signal magnitude and its spatial area, also contributed to the separation between classes. In comparison, the other three features—minimum intensity, area, and density—were less significant in classifying the data, indicating an insufficient ability, due to either the level of background intensity or the cell size, to discriminate positive cases from negative ones. The results indicate that the rare-earth photoluminescent signal is highly localized spatially and that it is also strongly driven by the intensities at each focal plane. The results further indicate that intensity-based features are reliable predictors for any automated classification schemes for nanoparticle–cell interaction experiments.

The results show that the model is solid and that the Random Forest method can reliably predict the presence of a photoluminescent signal in images of erythrocytes loaded with rare-earth nanoparticles. The model retained a good predictive ability on images of varying quality levels. We examined a total of 15 images with varying resolutions and signal-to-noise characteristics, and the model consistently returned positive ROIs across all images, indicating that it is relatively resistant to suboptimal imaging conditions. This suggests that the classifier could be useful in real-world microscopy workflows where uniformity in imaging cannot always be ensured.

**Figure 11:**
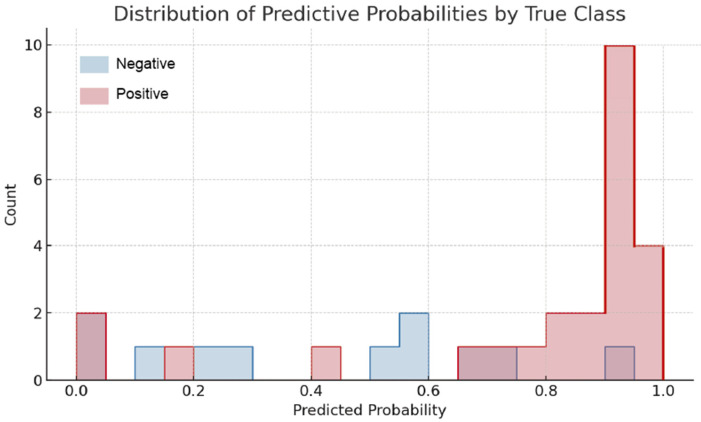
Histogram showing the distribution of predicted probability scores generated by the Random Forest model, separated by true class label. ROIs with a positive photoluminescent signal (red) are concentrated at high probability values (>0.85), while negative cases (blue) are distributed mostly below 0.15.

The trained Random Forest model produces predictive probability scores for each ROI to represent its confidence in each prediction, which ranged from 0 (negative prediction) to 1 (positive prediction). For the most part, the distribution of the predictive probabilities showed that most true positives were predicted with high confidence (>0.80) while most true negatives were predicted with predictive scores lower than 0.20 (Figure 11). This separation highlights the model’s ability to distinguish between classes using learned intensity patterns and further demonstrates its robustness.

Interestingly, a minor fraction of ROIs resulted in intermediate probability values (0.40–0.60), which may be ambiguous in terms of classification. These borderline predictions could perhaps be related to weak or heterogeneous photoluminescence signals, as well as ROIs that were compromised by poor image quality or marginal fluorescence. While this was a small fraction of all predictions, these cases are representative of biologically and technically relevant cases that could be highlighted for manual review or additional orthogonal analyses.

## 4. Discussion

The successful application of nanoparticle-based probes in bioimaging hinges on the precise control of their physicochemical properties and a thorough understanding of their interactions within complex biological environments. In this study, we synthesized and characterized NaYF_4_:Yb^3+^,Er^3+^ NPs and systematically optimized the conditions for their use in visualizing human erythrocytes via upconversion luminescence. The initial characterization confirmed the synthesis of monodisperse nanoparticles (average diameter of 29 ± 3 nm) possessing the desired hexagonal crystal phase (β-NaYF_4_), consistent with previous reports on optimized synthesis protocols for highly luminescent upconversion nanoparticles (UCNPs) [16,17]. Crucially, the nanoparticles exhibited the characteristic photoluminescence spectrum under 980 nm excitation, with prominent green (^2^H_11/2_,^4^S_3/2_→^4^I_15/2_ transitions) and red (^4^F_9/2_→^4^I_15/2_ transition) emission bands typical of Er^3+^ doping and efficient Yb^3+^ sensitization [18,19,20,21]. The high phase purity, confirmed by the absence of impurity peaks in the XRD pattern [22], is essential for maximizing the luminescence efficiency and ensuring reproducible optical behaviors.

A critical step for biological application was the surface modification, replacing the hydrophobic oleate ligands with hydrophilic polyacrylic acid (PAA). This ligand exchange serves a dual purpose, enhancing colloidal stability in aqueous media and providing functional groups that mediate interactions with cellular interfaces, while also potentially protecting the nanoparticle core from degradation induced by ions present in biological buffers [23]. The FT-IR analysis confirmed the successful coating of the NPs with PAA, evidenced by the characteristic vibrational bands of carboxyl and carboxylate groups, with the latter suggesting effective chelation onto the nanoparticle surface [24,25]. The resulting negative zeta potential (around −30 mV) is consistent with the presence of deprotonated carboxyl groups at physiological pH and is expected to influence electrostatic interactions with the negatively charged erythrocyte surface while also contributing to particle dispersion through mutual repulsion [26]. The thermogravimetric analysis further corroborated the presence of the organic PAA layer, quantifying it relative to the stable inorganic core.

Biocompatibility is of utmost importance for any material intended for cellular interaction. Our assessments using MTT assays on Vero cells and hemolysis assays on human erythrocytes revealed concentration-dependent effects. While the PAA coating likely improved the biocompatibility compared to uncoated or differently functionalized NPs, significant cytotoxicity and hemolysis were observed at higher concentrations (≥2.5 mg/mL). These findings align with previous studies demonstrating that rare-earth nanoparticles can exhibit toxicity at elevated doses [27,28,29]. However, at lower concentrations (≤1.0 mg/mL), particularly at the 0.1 mg/mL level, the cell viability remained high and hemolysis was minimal, suggesting acceptable biocompatibility for imaging applications within this range, consistent with other reports [30]. This necessitated careful selection of working concentrations (below 1.0 mg/mL) to balance the signal intensity requirements with minimizing adverse cellular effects.

The systematic optimization of experimental parameters for imaging erythrocytes proved crucial for achieving high-quality and reproducible results. The choice of buffer significantly impacted both nanoparticle stability and cell integrity. Our findings highlight the suitability of 100 mM HEPES buffer. Lower concentrations failed to maintain erythrocyte osmotic stability, leading to lysis, while higher concentrations introduced confounding autofluorescence [31,32], potentially obscuring the specific UCNP signal. Importantly, HEPES avoids issues associated with phosphate- or complex component-containing buffers like PBS or RPMI, which can reportedly lead to nanoparticle degradation or alterations in optical properties [23,33,34]. Our results confirm HEPES as a suitable alternative that preserves both NP and cell integrity under the tested conditions [33,35,36].

Incubation time emerged as another critical factor, influencing both the signal intensity and cell morphology. An optimal duration of 4 h maximized the photoluminescence signal, presumably reflecting the peak association or uptake of NPs by the erythrocytes, without inducing the significant morphological alterations (surface contouring and spherocytosis) observed at longer time points (6 and 24 h). This time-dependent effect on morphology is consistent with the literature suggesting that prolonged nanoparticle exposure can induce cellular stress, membrane damage, or structural changes [30,37,38]. The 4-h time point represents an optimal window, aligning with previous findings that intermediate incubation periods often yield the best interaction efficiency without compromising cell viability [37].

The optimization of optical parameters, namely laser current intensity and nanoparticle concentration, was essential for signal fidelity. A current intensity range of 250–300 mA provided sufficient excitation for bright upconversion luminescence without causing signal saturation or photothermal damage that can occur at higher intensities, particularly since 980 nm excitation is known to interact with water [39,40]. Similarly, an NP concentration of 0.01 mg/mL offered the best compromise between signal strength and biological impact. At this concentration, a bright signal, predominantly localized at the erythrocyte periphery, was achieved with minimal morphological disruption. Higher concentrations led to more intense but diffuse signals and significant cell deformation and aggregation, suggesting that excessive nanoparticle accumulation on the membrane surface can compromise its structural integrity. The peripheral localization observed at 0.01 mg/mL implies that the primary interaction under these conditions is adsorption onto the cell membrane rather than extensive internalization. This interaction pattern is plausible given the electrostatic repulsion expected between the negatively charged PAA coat and the erythrocyte glycocalyx, potentially limiting internalization but allowing for surface binding. Notably, the ability to detect a signal even with an UCNP concentration of 0.0001 mg/mL underscores their sensitivity as probes [41].

Additionally, the assessment of photostability under continuous laser exposure indicated that while UCNPs are generally more stable than conventional fluorophores, significant signal degradation can occur with prolonged irradiation times (>10 min). This necessitates limiting exposure during image acquisition to ensure quantitative accuracy and prevent potential photodamage to the nanoparticles or the cells themselves, which could arise from a reduced upconversion efficiency or laser-induced cellular stress that affects membrane permeability.

On the other hand, the use of supervised machine learning to analyze photoluminescent signals in erythrocyte images allowed for accurate and reproducible classification of nanoparticle–cell interactions. The Random Forest model produced good results with 80% accuracy, 83% precision, 81% recall, and an AUC-ROC of 0.85. These performance metrics suggest that the model is both sensitive and specific. Therefore, the model can detect the presence of rare-earth nanoparticle signals in complex biological images. The robust detection of photoluminescent ROIs both validated this experimental labeling strategy and demonstrated the strongly characteristic optical signature for the NaYF_4_:Yb^3+^,Er^3+^ nanomaterial used here. The analysis showed that intensity-based ROI-level features of the positive and negative classes exhibited the largest differences. ROIs containing a photoluminescent signal had significantly higher mean intensity, maximum intensity, and integrated density values, indicating good separation between the signal and no-signal classes. Importantly, these intensity-based variables also appeared to be the most significant inputs in the classifier’s decision-making process based on the three highest feature importance scores. Together, these results suggest that intensity-based features are both biologically relevant and informative for training an automatic signal detector.

Although the performance was good, some ROIs were still misclassified. Scrutiny of the predictive probabilities showed that most false positive and false negative cases had nearly borderline intensity values and an ambiguous morphology, reflecting the inherent challenges of visualizing low-signal or low-contrast structures. The fact that “blank” ROIs from the background were used as controls, implying a strong constraint against false positives, strengthens our confidence in the generalizability of the classifier. Future applications of this method would benefit from the use of predictive probability in flagging uncertain or difficult cases for manual analysis.

## 5. Conclusions

This work conclusively demonstrated that the optimized conditions significantly enhance the photoluminescence signal of NaYF_4_:Yb^3+^,Er^3+^@PAA-NPs interacting with erythrocytes. The identified optimal parameters—0.01 mg/mL nanoparticle concentration, a 4-h incubation period, the use of 100 mM HEPES buffer, and image acquisition within ten minutes—resulted in the maximum brightness and appropriate signal definition while preserving the morphological integrity of the red blood cells. Furthermore, our observations revealed that absorbed nanoparticles are predominantly located near the cell membrane. Importantly, the integration of machine learning methodologies for microscopic image processing and analysis significantly improved the image quality through enhanced pre- and post-processing techniques. This led to a more accurate and reproducible quantification of the nanoparticle–red blood cell interactions, bolstering the reliability of our findings. Collectively, these data provide a robust framework for optimizing the design of nanoparticle-based imaging systems and firmly establish the potential of NaYF_4_:Yb^3+^,Er^3+^@PAA-NPs as a valuable tool for bioimaging red blood cells.

## Figures and Tables

**Table 1:** Different experimental conditions used to enhance the RBC photoluminescence images.

Condition	Variation
HEPES concentration	10, 50, 100, 150, and 200 mM
Incubation time	1, 3, 4, 6, and 24 h
Photoluminescence detection limit of NaYF_4_:Yb^3+^,Er^3+^-NP concentration *	0.0001–1 mg/mL
Laser current intensity	50–450 mA
Exposure time	0, 5, 10, and 20 min

* The photoluminescence detection limit of the nanoparticles was determined through serial dilutions using the same microscope settings for this study.

## Data Availability

Data are contained within the article and Supplementary Materials.

## Supplementary Materials

The following supporting information can be downloaded at: https://www.mdpi.com/article/10.3390/bios15070396/s1, Figure S1: FT-IR and TGA analysis confirming the surface modification of nanoparticles with PAA; Figure S2: Boxplots showing the distribution of the integrated density for ROIs classified as negative or positive; Figure S3: Receiver operating characteristic (ROC) curve of the Random Forest model trained on ROI-level intensity features; Table S1: Quantitative analysis of brightness and definition of photoluminescence images of erythrocytes incubated with NaYF4:Yb^3+^,Er^3+^@PAA nanoparticles at different incubation times (1, 3, 4, 6, and 24 h).

## Abbreviations

The following abbreviations are used in this manuscript:

NPs	Nanoparticles
RBCs	Red blood cells
UCNPs	Upconversion nanoparticles
ML	Machine learning
RE-NPs	Rare-earth nanoparticles
TEM	Transmission electron microscopy
MTT assay	3-(4,5- dimethyl-2-thiazolyl)-2,5-di-phenyl-2-H-tetrazolium bromide
XRD	X-ray diffraction
FT-IR	Fourier-transform infrared spectroscopy
TGA	Thermogravimetric analysis
