# Supplementary material for: Enhanced Visualization of Erythrocytes Through Photoluminescence Using NaYbF4:Yb3+,Er3+ Nanoparticles
Source: Biosensors (Basel). 2025 Jun 20;15(7):396. doi: 10.3390/bios15070396 (PMC12292841; doi:10.3390/bios15070396)
Supplement: Supplementary file 1 [file biosensors-15-00396-s001.zip › biosensors-3676250-supplementary.pdf]

Enhanced Visualization of Erythrocytes Through Photoluminescence using $NaYbF_4:Yb^{3+},Er^{3+}$ Nanoparticles

Vivian Torres-Vera, Lorena M. Coronado, Ana Patricia Valencia, Alejandro Von Chong, Esteban Rua, Michelle Ng, Jorge Rubio-Retama, Carmenza Spadafora and Ricardo Correa

SUPPLEMENTARY INFORMATION

S1

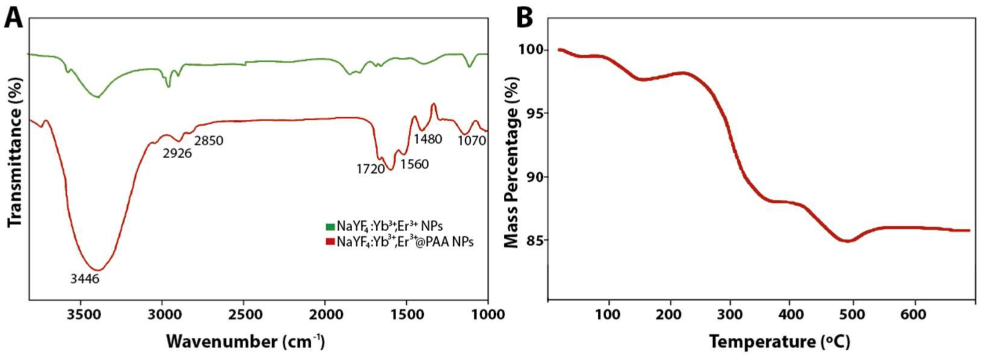

Figure S1. **A.** FT-IR spectra of $NaYF_4:Yb^{3+},Er^{3+}$ nanoparticles (green) and $NaYF_4:Yb^{3+},Er^{3+}$@PAA nanoparticles (red) showing the characteristic functional groups introduced by the PAA coating. **B** TGA curve of $NaYF_4:Yb^{3+},Er^{3+}$@PAA nanoparticles illustrating the mass loss attributed to the decomposition of the PAA layer.

Table S1

Table S1. Quantitative analysis of brightness and definition of photoluminescence images of erythrocytes incubated with $NaYF_4:Yb^{3+},Er^{3+}$@PAA nanoparticles at different incubation times (1, 3, 4, 6, and 24 hours).

Incubation Time	Brightness (Average Intensity)	Definition (Edge Density)
1 hour	184.88	7.12
3 hours	151.49	3.16
4 hours	218.17	5.13
6 hours	118.06	10.95
24 hours	105.45	6.16

Figure S2

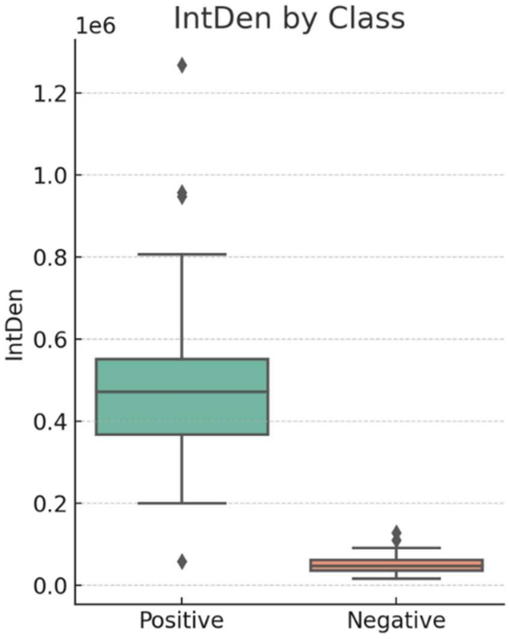

Figure S2. Boxplots showing the distribution of the integrated density for ROIs classified as negative and positive.

Figure S3

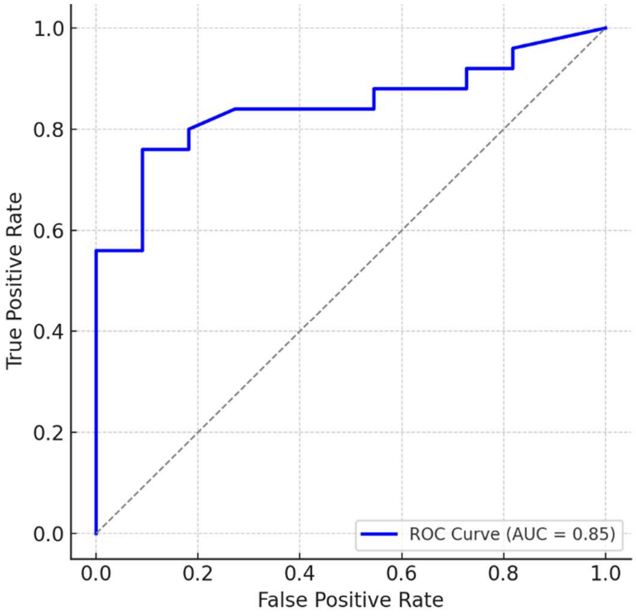

Figure S3. Receiver operating characteristic (ROC) curve of the Random Forest model trained on ROI-level intensity features.
